# Population pharmacokinetics and dose rationale for aciclovir in term and pre‐term neonates with herpes

**DOI:** 10.1002/prp2.1193

**Published:** 2024-05-22

**Authors:** S. D'Agate, D. Ruiz Gabarre, O. Della Pasqua

**Affiliations:** ^1^ Clinical Pharmacology & Therapeutics Group University College London London UK; ^2^ Present address: Institute for Regeneration and Repair University of Edinburgh Edinburgh UK

**Keywords:** aciclovir, dose rationale, *Herpes simplex*, neonates, pediatric extrapolation, pharmacokinetic modeling

## Abstract

Aciclovir is considered the first‐line treatment against *Herpes simplex* virus (HSV) infections in new‐borns and infants. As renal excretion is the major route of elimination, in renally‐impaired patients, aciclovir doses are adjusted according to the degree of impairment. However, limited attention has been given to the implications of immature renal function or dysfunction due to the viral disease itself. The aim of this investigation was to characterize the pharmacokinetics of aciclovir taking into account maturation and disease processes in the neonatal population. Pharmacokinetic data obtained from 2 previously published clinical trials (*n* = 28) were analyzed using a nonlinear mixed effects modeling approach. Post‐menstrual age (PMA) and creatinine clearance (CL_CR_) were assessed as descriptors of maturation and renal function. Simulation scenarios were also implemented to illustrate the use of pharmacokinetic data to extrapolate efficacy from adults. Aciclovir pharmacokinetics was described by a one‐compartment model with first‐order elimination. Body weight and diagnosis (systemic infection) were statistically significant covariates on the volume of distribution, whereas body weight, CL_CR_ and PMA had a significant effect on clearance. Median clearance varied from 0.2 to 1.0 L/h in subjects with PMA <34 or ≥34 weeks, respectively. Population estimate for volume of distribution was 1.93 L with systemic infection increasing this value by almost 3‐fold (2.67 times higher). A suitable model parameterization was identified, which discriminates the effects of developmental growth, maturation, and organ function. Exposure to aciclovir was found to increase with decreasing PMA and renal function (CL_CR_), suggesting different dosing requirement for pre‐term neonates.

## Graphical abstract

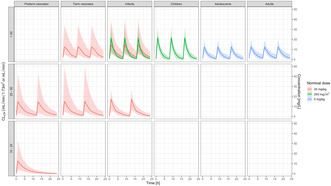

Predicted steady‐state plasma aciclovir concentration vs. time profiles after administration of recommended doses of aciclovir (i.e., 20 mg/kg, 250 mg/m^2^ and 5 mg/kg) to paediatric and adult patients, stratified by creatinine clearance.

These profiles show that the current regimen for preterm neonates results in lower daily exposure to aciclovir, as compared to children, adolescents and adults.

**Abbreviations**
AKI: acute kidney injury
AUC: area under the concentration versus time curve
BSA: body surface area
CL: clearance
CL_CR_
: creatinine clearance
*C*_max_
: maximum concentration
CMV: cytomegalovirus
CWRES: conditional weighted residuals
*df*
: degree of freedom
EBV: Epstein–Barr virus
GA: gestational age
GFR: glomerular filtration rate
HSV: *Herpes simplex* virus
IIV: interindividual variability
NPDE: normalized prediction distribution error
OFV: objective function value
PBPK: physiologically‐based pharmacokinetic
PK: pharmacokinetics
PMA: post‐menstrual age
PNA: post‐natal age
RSE: residual standard error
Scr: serum creatinine
SVPC: standardized visual predictive check
V: volume of distribution
VPC: visual predictive check
VZV: *Varicella zoster* virus
WT: body weight

## 1 INTRODUCTION

*Herpes simplex* virus (HSV) infection is uncommon amongst neonates, with an overall incidence of approximately 9.6 cases per 100 000 live births.^1^ HSV infection can manifest in different forms of in neonates, including skin, eye, mouth, CNS and systemic infection, with varying degrees of severity. Even though the clinical management of subjects potentially exposed to HSV during delivery is common practice, early diagnosis, and prompt pharmacological intervention with antiviral drugs, such as aciclovir, are critical steps to prevent potential sequelae from infection.

Aciclovir is a synthetic purine nucleoside analogue with inhibitory activity against human HSV types 1 and 2, *Varicella zoster* virus (VZV), *Epstein Barr* virus (EBV) and cytomegalovirus (CMV). It has been available for clinical use for over three decades and has demonstrated remarkable safety and efficacy against mild to severe infections in both normal and immunocompromised patients.^2^ The initial aciclovir treatment regimen for neonatal HSV disease was empirically chosen to be 30 mg/kg/day given intravenously for 10 days.^3^ However, given existing concerns about the persistence of HSV in the CNS at the end of therapy, the use of a higher dose and a longer duration of therapy (45–60 mg/kg/day up to 21 days) has been proposed. Subsequently, an open‐label study evaluating higher doses of aciclovir for neonatal HSV infections showed that a dose of 60 mg/kg/day for 14–21 days decreased mortality compared to 30 mg/kg/day for 10 days.^4^ A systematic review by Jones et al. found that despite a preference for the increased dose and duration of aciclovir therapy, no clinical trials exist that provide more robust evidence.^5^ In addition, more recent studies have concluded that although clinical and laboratory AEs were common in infants treated with high (>60 mg/kg/day) and very high (>80 mg/kg/day) doses aciclovir, these were not severe. Moreover, a relationship between aciclovir exposure and incidence of AEs could not be established.^6^, ^7^ All things considered, it has been shown that high dose aciclovir yielded serum steady‐state concentrations above the pharmacodynamic target and maximum concentrations below the safety target.^7^ Consequently, the currently recommended regimen for treatment of known or suspected neonatal herpes has been defined as 20 mg/kg aciclovir every 8 h (i.e., 60 mg/kg/day) for 21 days for disseminated and CNS disease, or for 14 days if the disease limited to the skin and mucous membranes.^8^

Such a rationale raise an interesting point, namely, that if aciclovir had been developed according to current guidelines for pediatric drug development,^9^, ^10^ its efficacy in neonates could have been extrapolated from adults and older pediatric patients based on a dosing regimen that yields exposure comparable to the efficacious range observed in the reference population. Yet, there has not been any systematic attempt to establish how exposure in neonates compares across the overall population.^11^, ^12^ Besides, available pharmacokinetic models do not include the effect of disease and organ function to better understand interindividual differences in exposure and consequently establish the dose rationale for this age group.^13^ Of note has been the lack of covariates that describe the maturation of renal function (i.e., glomerular filtration and active tubular secretion) over the first few weeks after birth.

Renal excretion by passive glomerular filtration is the major route of elimination of aciclovir, but active tubular secretion also contributes to renal clearance.^14^, ^15^ It has been shown in adults and children that total clearance (CL) and half‐life are dependent on renal function, as evaluated by estimated creatinine clearance (CL_CR_). Therefore, patients with impaired renal function require an appropriately modified dose, according to the degree of impairment. While dosing recommendations for neonatal infections, which take into account the degree of impairment are found in different guidelines,^16^ limited attention has been given to the potential implications of immature renal function or renal dysfunction associated with the progression of viral disease itself.^17^, ^18^ There is evidence that aciclovir elimination in neonates, and in particular those who are born pre‐term, is slower due to immature glomerular filtration rate (GFR) and reduced tubular function.^19^ Such a slower elimination appears to reflect the lower rate of increase in GFR in pre‐term neonates, as compared to those born at term.^20^

From a clinical perspective, neonatal patients receiving aciclovir should be exposed to efficacious and safe drug concentrations, irrespective of individual differences in age, body weight or organ function. In fact, exposure should be similar across different age groups, given that viral load has been shown to vary within the same range in both adult and pediatric populations.^21^, ^22^ Consequently, the dose rationale in this group of patients should take into account the influence of (patho)physiological factors that can affect pharmacokinetics. Indeed, previous investigations with other antibiotics have shown the importance of organ maturation and implications of disease‐related changes in drug disposition in pre‐term and term neonates.^23^, ^24^, ^25^ Hence, it would be important to characterize the implications of variable renal function and disease on the pharmacokinetics of aciclovir in this population.

Despite the availability of a model describing the disposition of aciclovir in this patient population,^26^ the contribution of different covariate factors has not been fully characterized. Disposition parameters (CL and V) were assumed to be linearly related to body weight, with highly correlated estimates between CL and V (correlation coefficient .98), which exceeds the usual physiological correlation between these parameters. Interindividual differences were explained by post‐menstrual age (PMA) and stochastic components without considering renal function. The aim of this analysis was therefore to re‐parameterize the pharmacokinetic disposition of aciclovir to include the effect of maturation processes and renal function in neonatal patients with or without suspected systemic infection. The model is subsequently used to assess the effect of interindividual variability on systemic exposure and illustrate how the efficacy of antiviral drugs such as aciclovir in preterm and term neonates could be inferred from evidence of matching exposure across age groups, i.e., children, adolescents and adults.

## 2 METHODS

### 2.1 Pharmacokinetic data

The population pharmacokinetics of aciclovir in neonatal patients (*n* = 32) was characterized based on the data from Sampson and colleagues. The data set consisted of two studies. Study 1 was a single‐center, open label, pharmacokinetic study of infants at 23–42 weeks gestational age and <61 days postnatal age with suspected systemic infection, whereas study 2 was a multicenter, open label, pharmacokinetic study of infants at 23–34 weeks gestational age and <45 days postnatal age with suspected systemic HSV infection. Further details about the studies are available in Table S1.^26^ Model development was focused on identifying opportunities to refine the model reported previously for the clinical study (Table S2). Attention was given to an alternative model parameterization, which enabled us to disentangle the effect of size, maturation processes (age) and organ function on clearance. In addition, we evaluated the role of disease on drug disposition by treating suspected systemic infection as a covariate factor.

### 2.2 Model development

General model building criteria were applied to ensure that a suitable structural PK model could be identified first. This step was followed by introducing the appropriate stochastic model describing between‐subject variability. Selected covariates were then added to the base model according to a stepwise forward addition‐backward elimination procedure. Comparison of hierarchical models was based on the likelihood ratio test and the standard error of the parameter estimates. A covariate analysis was performed to explore identifiable sources of pharmacokinetic variability for aciclovir. The following demographic and clinical baseline covariates were considered for the analysis: age, sex, race, weight at birth and weight, serum creatinine, use of concurrent medication (vasopressin, epinephrine, dopamine), disease severity (i.e., absence or evidence of systemic infection).

Covariate model building was conducted in a stepwise manner and the likelihood ratio was used to test the effect of each covariate on model parameters with a significance level of 0.01. In the step‐wise forward addition procedure, a covariate was considered significant if the reduction in the objective function value (OFV) between the base and more complex model was greater than 3.84 (*χ* ^2^ < 0.05 for 1 degree of freedom, *df*). All significant covariates were then added simultaneously into the full model. Subsequently, each covariate was independently removed from the full model if the increase in the OFV was less than 6.64 (*χ* ^2^ < 0.01 for 1 *df*). Otherwise, the covariate was considered to be significantly correlated with the pharmacokinetic parameter and retained in the final model.

First, allometric scaling and maturation concepts were applied to characterize the effect of body size and developmental growth on the clearance of aciclovir. It has been widely established that body weight describes the effect of body size on the disposition properties of most drugs, also when maturation processes co‐exist.^27^, ^28^, ^29^ A sigmoidal function was used to describe the contribution of maturation processes based on the assumption that PMA can be considered a proxy for maturation‐related changes in renal clearance.^27^ Second, the implications of varying organ function due to the disease itself or other pathological conditions were also considered during model refinement. Although the correlation between organ function and developmental growth may not be easily derived from the data, previous investigations suggest that organ function can be described by creatinine clearance using the Schwartz formula.^30^, ^31^

In addition, overall higher concentration profiles were observed for Study 1 compared to Study 2 (Table S1). After further data exploration for the effect of potential covariates, it became evident that such differences may reflect changes in the volume of distribution. Systemic infection, in fact, has been previously shown to alter drug distribution.^32^ Given the different proportion of positive virological findings in blood samples,^33^ it is conceivable, therefore, that the degree of systemic infection might differ between subjects in the two studies.

### 2.3 Covariate model building

Different approaches have been considered to evaluate the covariate effect on the pharmacokinetic parameters of interest, in particular, the changes in clearance. These approaches included the following:

1. the use of pre‐defined fixed allometric exponents (0.75 for CL).
2. the use of a maturation function based on the previous publication by Sampson et al.^26^ in which the combination of a maturation function and allometric scaling describes drug disposition in neonates and young infants. Typical values for clearance, were defined as follows:

$$\text{TVCL}=\theta_{\mathrm{CL}}\times {\left(\frac{\mathrm{WT}}{1.37}\right)}^{0.75}\times {\left(\frac{\mathrm{PMA}}{31.3}\right)}^{\theta_{\mathrm{PMA}}}$$

where PMA is the post‐menstrual age in weeks, 31.3 is the median post‐menstrual age in the population and $\theta_{\mathrm{PMA}}$ is the estimated exponent for post‐menstrual age. WT is the weight in kg and 1.37 kg is the median weight for the population.

3 Estimation of residual clearance in combination with the calculation of creatinine clearance. The Schwartz formula was used for the calculation of the creatinine clearance:

$${\mathrm{CL}}_{\mathrm{CR}}=\mathrm{HT}\times \frac{k}{\mathrm{SCr}}$$

where *k* = 0.33 if gestational age is less than 36 weeks and *k* = 0.45 otherwise. HT is the height of the subject in cm and SCr is the serum creatinine concentration in mg/L. SCr was measured multiple times for each subject; so, CL_CR_ was introduced as a time‐varying covariate. The values obtained were converted from mL/min/1.73 m^2^ to L/h using the individual body surface area of the subject as calculated with Gehan and George formula.^34^

The total clearance was calculated as follows:

$$\text{TVCL}=\theta_{\mathrm{CL},\mathrm{res}}\times {\left(\frac{\mathrm{WT}}{1.37}\right)}^{0.75}\times \left(\frac{\mathrm{PM}A^{\text{HILL}}}{{\mathrm{PMA}}_{50}^{\text{HILL}}+\mathrm{PM}A^{\text{HILL}}}\right)+{\mathrm{CL}}_{\mathrm{CR}}$$

where $\theta_{\mathrm{CL},\mathrm{res}}$ represents the residual clearance that is not explained by the changes in creatinine clearance. WT is the weight in kg and 1.37 kg is the median weight for the population. PMA is the post‐menstrual age in weeks, HILL is the shape factor for the maturation formula and PMA_50_ is the time at which the maturation process reaches half of the maximum value.

The effect of body weight on *V* was evaluated considering a pre‐defined fixed allometric exponent with value 1. In addition, given the high interindividual variability in *V*, further steps were taken to assess the effect of disease severity (i.e., systemic infection), using study as a discrete covariate.

### 2.4 Model evaluation

Goodness‐of‐fit was assessed by graphical methods, including population and individual predicted versus observed concentrations, conditional weighted residuals versus observed concentration or time, and the correlation between parameters. In addition to a typical visual predictive check, standardized visual predictive check (SVPC) was used to evaluate the adequacy of the final model parameter estimates, including the effects of statistically significant covariates to produce simulated data that were similar to the original observed data. The SVPC was implemented to better distinguish model misspecification from the effect of different study designs or sparse sampling of the data.^35^ According to this method, the differences between the observed and simulated values are caused only by structural model misspecification and/or inadequate estimation of the inter‐ and intra‐subject variability. One thousand replicates of the original data set were simulated, based on the final model, and the percentile of each participant in the marginal distribution of the simulated endpoint as a function of time (or any covariate of interest) calculated, so that each subject design template (e.g., dose, dosing schedule, values of influential covariates) was taken into account.

Bootstrapping was performed to identify bias, stability, and accuracy of the parameter estimates, generate standard errors and confidence intervals. Perl‐speaks‐NONMEM (PsN) was used to generate 2000 new data sets by sampling individuals with replacement from the original one and then fitting the model to each new data set.

Despite the limited number of patients included in this analysis, further evaluation of the variance–covariance structure and overall random effects in the model was performed using mirror plots and NPDE diagnostics. To generate mirror plots, the population PK parameters estimates were used to simulate plasma concentrations in patients with similar demographic characteristics, dosing regimens, and sampling scheme as the original clinical studies. Mirror plots of individual predicted versus observed concentration were created to evaluate the degree of similarity between the original fit and the pattern obtained from the simulated data sets. Finally, the normalized prediction distribution error (NPDE) was estimated. Plots to evaluate whether the discrepancies between observed and predicted values were normally distributed included a histogram of the NPDE with the density of the standard normal distribution overlaid, a scatter plot of the NPDE versus observed values, and a scatter plot of NPDE versus predicted values.

### 2.5 Aciclovir exposure in neonates, children, adolescents and adults

Even though aciclovir has been approved for pediatric use for more than three decades, its approval was based on limited, empirical evidence of efficacy and safety in neonatal patients. Current guidelines have evolved, including dose rationale and requirements for generating efficacy data in children when the same indication or condition exists in adults. Aciclovir represents, therefore, a case example for the use of extrapolation principles. To illustrate how pharmacokinetic data could be used as basis for the extrapolation of efficacy in adults, simulation scenarios were implemented in a virtual cohort of preterm and term new‐borns, infants, children, adolescent and adult subjects (*N* = 100 per cohort), whose systemic exposure was characterized following different dosing regimens. Each cohort received doses as reported in the summary of product characteristics^8^: neonates (20 mg/kg t.i.d.), infants (20 mg/kg t.i.d. <3 months and 250 mg/m^2^ t.i.d. ≥3 months), children (250 mg/m^2^ t.i.d.), adolescents (5 mg/kg t.i.d.) and adults (5 mg/kg t.i.d.). Similarly, dose adjustment was based on creatinine clearance as reported in the summary of product characteristics:

- CL_CR_ = 25 to 50 mL/min: dose administered every 12 h
- CL_CR_ = 10 to 25 mL/min: dose administered every 24 h
- CL_CR_ = 0 (anuric) to 10 mL/min: dose halved and administered every 24 h

Doses were adjusted according to BSA‐normalized CL_CR_ (mL/min/1.73 m^2^) for neonates, infants, children, and adolescent and non‐normalized CL_CR_ (mL/min) for adults.

Secondary pharmacokinetic parameters at steady state, including the area under the concentration versus time curve (AUC_ss0‐24_) and maximum concentration (*C*max_ss_), were derived as metrics of exposure to aciclovir. Data were then summarized for preterm and term neonates, infants, children, adolescents and adults using covariate distributions obtained from the NHANES and CALIPER databases. For subjects aged less than 2 years, creatinine clearance was calculated using the Schwartz formula with exponent *k* = 0.33 if gestational age was less than 36 weeks and *k* = 0.45 otherwise,^36^ whereas for subjects aged between 2 and 18 years the exponent used was *k* = 0.413.^37^ Creatinine clearance in adults was calculated using the Cockcroft‐Gault formula.^38^ A previously published pharmacokinetic model by Zeng et al. was used for the simulation of pediatric and adult subjects older than 3 months post‐natal age.^29^ For completeness, model estimates are reported in the Supporting Information (Table S3). Secondary parameters were stratified by indication, dose and body weight where appropriate. Data were summarized in graphical and tabular format using descriptive statistics.

## 3 NOMENCLATURE OF TARGETS AND LIGANDS

Key protein targets and ligands in this article are hyperlinked to corresponding entries in http://www.guidetopharmacology.org, the common portal for data from the IUPHAR/BPS Guide to PHARMACOLOGY,^39^ and are permanently archived in the Concise Guide to PHARMACOLOGY 2019/20.^40^

## 4 RESULTS

Ninety‐two plasma samples from 32 infants were collected in the original clinical studies. An overview of patient demographics is reported in Table 1. Nine (9.8%) samples were excluded prior to model development, as they were previously considered contaminated or drawn during infusion. Concentration data from 4 samples were identified as potential outliers during the check out and visual examination of individual and pooled PK profiles. The samples were nevertheless included in the final analysis since they did not appear to have significant impact on parameter estimates. The final data set included 83 samples from 28 infants.

**TABLE 1:** Summary of demographics and clinical baseline characteristics of the patients included in the clinical studies (*N* = 32).

	*N* or Median (range)
GA (weeks)	30 (23–40)
PMA (weeks)	31 (25–41)
PNA (days)	3 (1–30)
Birth weight (g)	1295 (420–4840)
Weight (g)	1420 (373–5720)
Female/Male	17/15
White/Black/Asian	20/11/1
SCr (mg/dL)	0.9 (0.3–1.8)
Vasopressin use	1
Dopamine use	4
Epinephrine use	7

### 4.1 Pharmacokinetic modeling

The final model was a one‐compartment open model with zero‐order infusion and first‐order elimination. Interindividual variability (IIV) terms were identified for clearance (CL) and volume of distribution (V). Body weight, PMA, and CL_CR_ were found to be statistically significant covariates on aciclovir clearance. Furthermore, the inferred effect of systemic infection on the volume of distribution was found to be significant. The use of study as a proxy for the disease status provided a suitable alternative to the lack of individual details on baseline viral load. Fixed effect parameters and IIV estimates showed good precision (RSE < 39% and RSE < 43%, respectively). In addition, all parameters were well estimated without significant correlations between them. An overview of the results is summarized in Table 2.

**TABLE 2:** Final pharmacokinetic model parameter estimates.

Parameter (unit)^a^	Notation	Population estimate	RSE (%)	Bootstrap median (95% CI)
Systemic clearance, CL (L/h) = *θ*1*(WT/1.37)^0.75*(PMA/(*θ*3 + PMA)) + CLCR	*θ*1	0.748	18	0.730 (0.429–1.04)
	*θ*3	50^b^	–	–
Central volume of distribution, V (L) = *θ*2*(WT/1.37)*DIS*θ4	*θ*2	1.93	39	1.89 (0.823–3.50)
	*θ*4	2.67	38	2.74 (1.46–6.43)

Inter‐individual variability^c^		Population estimate (CV%)	RSE (%)	Bootstrap median (95% CI)
*η*CL variance	Ω1	0.451 (75.5%)	13	0.423 (0.198–0.693)
*η*V variance	Ω2	0.646 (95.3%)	23	0.624 (0.224–1.46)
*η*CL‐V covariance (ρCL‐V%)		0.442 (82%)	42.3	0.417 (0.147–0.939)

Residual error		Population estimate (CV%)	RSE (%)	Bootstrap median (95% CI)
Proportional error	*σ*1	0.143 (37.8%)	17	0.140 (0.07–0.250)

Abbreviations: CI, confidence interval; CLCR, creatinine clearance; CV, coefficient of variation; DIS, disease status; PMA, post‐menstrual age; RSE, relative standard error; WT, body weight; *η*, inter‐individual variability; *θ*, PK parameter estimation; *σ*, population variance; Ω, inter‐individual or inter‐occasion variability in population PK parameter.

^a^ Population parameter point‐estimates for the full one compartment model and 95% CI and %CV from a non‐parametric bootstrap are presented.

^b^ Fixed to literature value.

^c^ Value in parentheses represents the inter‐individual variability of the PK parameters calculated as the square root of (e^Ω^ − 1) × 100%.

**FIGURE 1:**
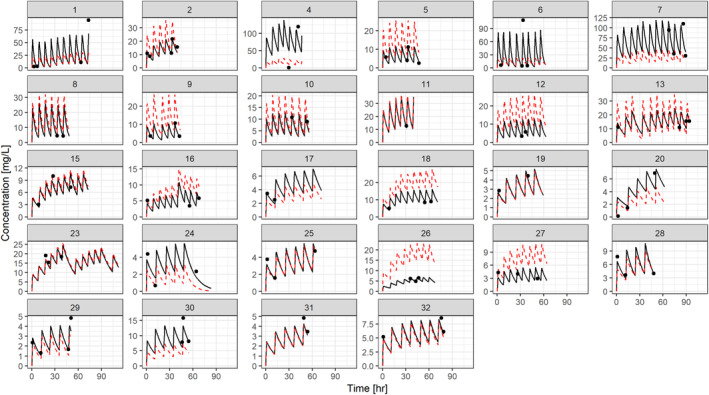
Individual aciclovir plasma concentration versus time plots. Panels show the individually fitted concentration versus time profiles as predicted by the final model. Black circles are the observed concentrations. Solid black and dotted red lines indicate the individually and population predicted concentrations, respectively.

Despite the large variability in the data, the diagnostic plots for the final model in Figure S1 show that the model was able to describe the data, yielding unbiased population and individual predictions. Predicted and observed concentration‐time profiles are shown by subject in Figure 1. Additionally, the stochastic parameter distribution describing interindividual variability was close to normal and uncorrelated. The CWRES scattering did not suggest bias or significant deviation between predicted and observed concentrations (Figure S1). No correlations or trends were noted between the conditional weighted residuals or body weight.

**FIGURE 2:**
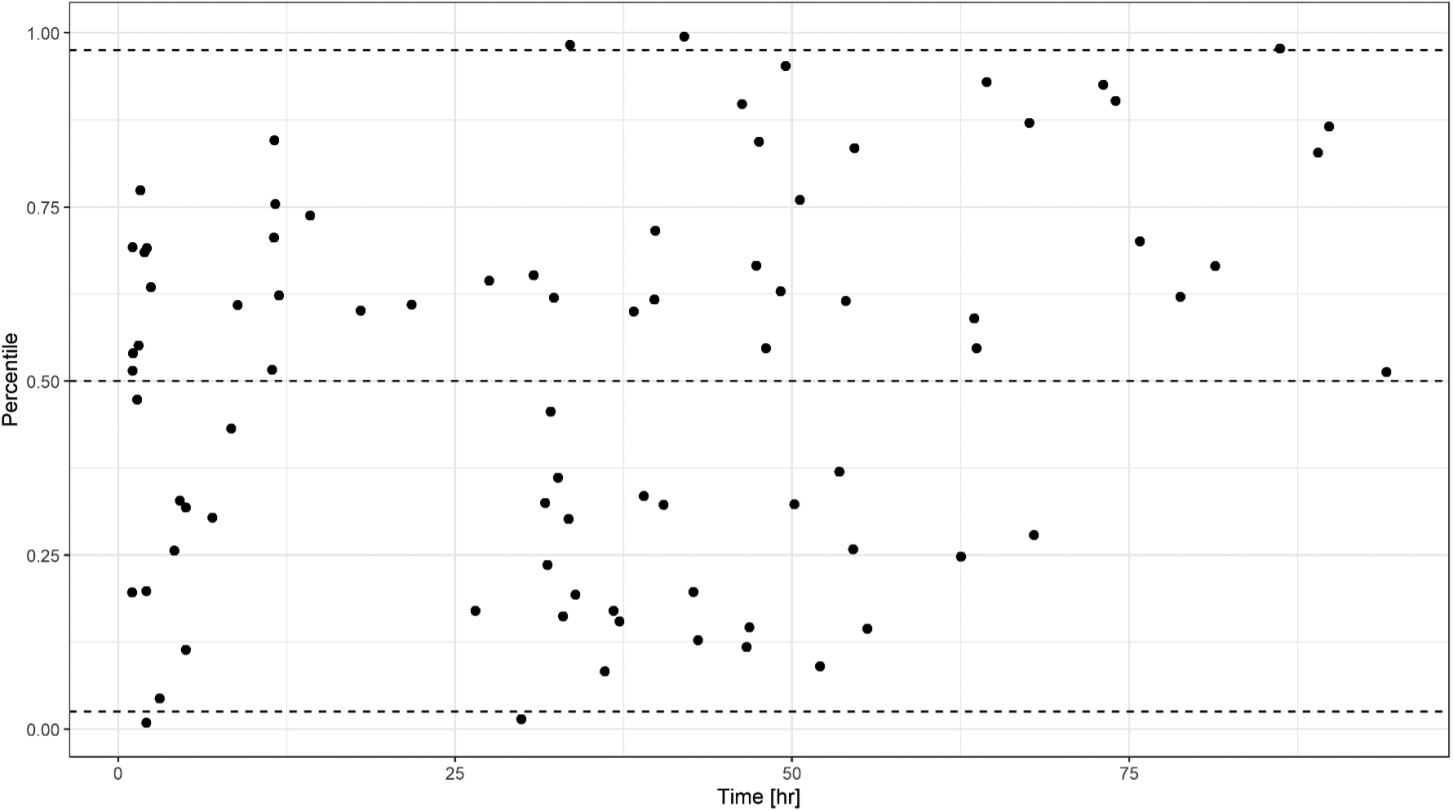
Standardized visual predictive check (SVPC) plots. SVPC represents the percentile of each subject observation in the distribution of simulated values at the same time point accounting for differences in dosing regimens, covariates, and sampling schedule between individuals. Black circles represent the calculated individual percentiles for each observation versus time. Dashed line represents the theoretical 5^th^, 50^th^ and 95^th^ percentiles of probabilities. Additional diagnostics showing typical VPCs are included in the Supporting Information (Figure S4).

Standardized VPC (Figure 2) showed that the observed concentrations fell within the 95% confidence intervals of the simulated values. In addition, the non‐parametric bootstrap estimates of the model parameters were similar to the final model estimates (Table 2). The mirror plots in Figure S2 indicate that the final model accurately replicates the profiles of aciclovir in this patient population. On the other hand, the NPDE plots displayed some deviation from the standard normal distribution for the prediction error but did not reveal any particular bias in model predictions following intravenous doses (Figure S3). This deviation may be partly caused by the small sample size. As exclusion of the data points which showed greatest deviation did not improve the goodness‐of‐fit, no data were excluded for the estimation of the final model parameters.

Based on the goodness‐of‐fit, as well as on the results from the bootstrap, SVPC and NPDE, the final model was deemed to have acceptable performance to describe aciclovir exposure in neonatal patients. It can be assumed that model structure and covariate effects associated with developmental growth, maturation processes and organ function are sufficiently robust for subsequent use of the model for simulation purposes.

The most notable difference between the original model published by Sampson and colleagues and the final estimated model was the effect of creatinine clearance and infection status or severity on disposition parameters. However, final parameter estimates did not differ significantly from published values.^26^ A comparison of the reported exposure to aciclovir, expressed as trough and peak concentrations at steady state, is presented in Table S4 along with the *post‐hoc* estimates obtained from the current analysis. Interestingly, based on the median [90%CI] calculated CL_CR_ and *post‐hoc* estimates of total clearance (i.e., 15.8 [9.4–40.0] and 71.0 [26.2–257.6] mL/min/1.73 m^2^, respectively), it appears that CL_CR_ accounts for approximately one fourth of the total clearance of aciclovir. These findings are summarized in Table S5, which shows the individual CL estimates and associated CL_CR_ for each subject, stratified by CL_CR_ and PMA.

### 4.2 Exposure in neonates, children, adolescents and adults

**FIGURE 3:**
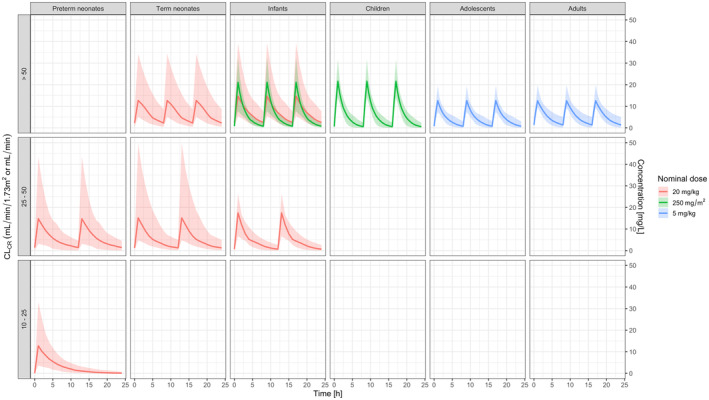
Steady‐state aciclovir concentration versus time profiles in (pre‐)term neonates, infants, children, adolescents, and adults. Each panel shows the pharmacokinetic profiles after intravenous administration of aciclovir to neonates (20 mg/kg t.i.d.), infants (20 mg/kg t.i.d. <3 months and 250 mg/m^2^ t.i.d. ≥3 months), children (250 mg/m^2^ t.i.d.), adolescents (5 mg/kg t.i.d.) and adult subjects (5 mg/kg t.i.d.) as reported in Table 3. Doses were adjusted according to BSA‐normalized CL_CR_ (mL/min/1.73 m^2^) for neonates, infants, children, and adolescents and non‐normalized CL_CR_ (mL/min) for adults. Lines represent the median simulated concentrations, shaded areas describe the 5^th^ and 95^th^ percentiles of the simulated concentration.

In Figure 3 an overview of the predicted aciclovir exposure after administration to neonates, infants, children and adolescents is compared with values obtained in adult subjects following the recommended dose for HSV infection.

The demographic and clinical baseline characteristics of the subjects included in the simulations are presented in Table 3, along with the median and 90% confidence intervals of the secondary pharmacokinetic parameters. As PMA is a covariate factor in the model describing the disposition of aciclovir in neonates, summaries were split into pre‐term (<37 weeks of gestational age (GA)) and term new‐borns (≥37 weeks GA).

**TABLE 3:** Predicted aciclovir exposure in (pre‐)term neonates, infants, children, adolescents, and adults, including the demographic characteristics of each simulated cohort.

	Preterm neonates (*N* = 100)		Term neonates (*N* = 100)		Infants (*N* = 100)		Children (*N* = 100)		Adolescents (*N* = 100)		Adults (*N* = 100)	
Characteristic	Median	Range	Median	Range	Median	Range	Median	Range	Median	Range	Median	Range
Gestational age (weeks)	27.44	[24.01,36.78]	39.43	[37,41.84]	31.39	[24.02,41.73]	37	[24.11,41.03]				
Post‐natal age (days)	8.89	[0.15,27.43]	14.67	[0.91,27.97]	65.37	[28.66,357.9]	1916.3	[373.26,4319.2]				
Post‐menstrual age (weeks)	29	[24.22,38.73]	41.71	[37.54,45.3]	41.06	[28.11,88.39]	310.75	[79.73,654.02]				
Age (years)	0.02	[0,0.08]	0.04	[0,0.08]	0.18	[0.08,0.98]	5.25	[1.02,11.83]	14	[12,17]	59	[18,84]
Weight (kg)	1.18	[0.32,5.59]	3.3	[1.28,8.38]	3.12	[0.51,15.54]	20.45	[5.28,54.8]	56.45	[31.8,92.6]	71.4	[48.6,100.6]
Length/Height (cm)	40.31	[36.67,58.97]	49.27	[40.72,70.78]	48.5	[37.46,101.06]	112.1	[57.65,160.5]	161.75	[141.2,193.1]	170.85	[144.3,189.7]
Body surface area (m^2^)	0.12	[0.06,0.32]	0.23	[0.13,0.42]	0.22	[0.08,0.68]	0.82	[0.31,1.58]	1.61	[1.13,2.2]	1.85	[1.45,2.3]
Serum creatinine (mg/dL)	0.6	[0.36,0.84]	0.49	[0.35,0.81]	0.25	[0.24,0.35]	0.37	[0.24,0.53]	0.7	[0.4,1.1]	0.9	[0.4,1.6]
CL_CR_ (mL/min/1.73m^2^)	23.53	[15.18,49.89]	46.2	[27.01,89.71]	67.35	[36.23,192.48]	124.28	[80.52,207.34]	96.23	[63.64,169.02]	86.99	[45.34,238.67]
Sex (F/M)	51/49		54/46		57/43		52/48		45/55		39/61	

**PK parameters**	**Median (95% CI)**	**Median (95% CI)**	**Median (95% CI)**		**Median (95% CI)**	**Median (95% CI)**	**Median (95% CI)**
**Dose**	**20 mg/kg**	**20 mg/kg**	**20 mg/kg**	**250 mg/m** ^**2**^	**250 mg/m** ^**2**^	**5 mg/kg**	**5 mg/kg**
CL_CR_ > 50 mL/min^a^							

**Total daily dose (t.i.d. regimen)**	**60 mg/kg**	**60 mg/kg**	**60 mg/kg**	**750 mg/m** ^**2**^	**750 mg/m** ^**2**^	**15 mg/kg**	**15 mg/kg**
AUC_0–24_	–	185 (59, 457)	176 (66, 417)	156 (90, 222)	153 (109, 220)	110 (64, 169)	134 (80, 206)
*C*_max_	–	12.7 (5.1, 34.4)	14.7 (5.4, 39.3)	21.2 (7.8, 32.9)	21.7 (14, 32)	12.7 (7.3, 19.6)	12.6 (9.3, 19.9)
CL_CR_ = 25–50 mL/min^a^							

**Total daily dose (b.i.d. regimen)**	**40 mg/kg**	**40 mg/kg**	**40 mg/kg**	**500 mg/m** ^**2**^	**500 mg/m** ^**2**^	**10 mg/kg**	**10 mg/kg**
AUC_0–24_	134 (37, 327)	134 (46, 434)	109 (65, 200)	–	–	–	–
*C*_max_	14.8 (3.2, 43.6)	15.2 (4.7, 49.8)	17.5 (6.7, 26.3)	–	–	–	–
CL_CR_ = 10–25 mL/min^a^							

**Total daily dose (q.d. regimen)**	**20 mg/kg**	**20 mg/kg**	**20 mg/kg**	**250 mg/m** ^**2**^	**250 mg/m** ^**2**^	**5 mg/kg**	**5 mg/kg**
AUC_0–24_	70 (24, 144)	–	–	–	–	–	–
*C*_max_	12.8 (3.6, 33.1)	–	–	–	–	–	–

^a^ Doses were adjusted according to BSA‐normalised CL_CR_ (mL/min/1.73m^2^) for neonates, infants, children, and adolescents and non‐normalised CL_CR_ (mL/min) for adults.

The main differences were in preterm neonates, for whom model‐predicted exposure to aciclovir (AUC_0–24_) was lower than in adults. By contrast, infants and children showed higher exposure than adults. In addition, within the neonatal group, term neonates show considerably higher AUC_0‐24_ than preterm neonates. However, this can be explained by the presence of preterm new‐borns who, due to a low creatinine clearance (<25 mL/min/1.73 m^2^), receive a lower total daily dose.

## 5 DISCUSSION AND CONCLUSIONS

The dose rationale for neonates, and in particular in pre‐term new‐borns, has been based on empirical evidence of efficacy and safety, without further consideration of the different factors that may determine drug disposition and overall exposure to the active moiety. Although pharmacokinetic data remains limited in the neonatal patient population, the current investigation aimed to evaluate the effect of developmental growth, organ function and disease severity on the disposition of aciclovir to better understand interindividual differences in exposure and consequently confirm the dose rationale for this age group based on pharmacokinetic‐pharmacodynamic principles.^11^, ^12^, ^13^ In addition, our analysis has shown how exposure in neonates compares with older infants, children, adolescents and adult patients.^14^, ^29^

Even though there is limited understanding of the mechanisms associated with the maturation processes and the effect of renal dysfunction on the clearance of aciclovir in pre‐term and term neonates, we believe that the current results provide further insight into the role of age‐ and disease‐related factors on the disposition properties in this small group of patients. A suitable covariate model was identified, which discriminates between the changes associated with developmental growth, maturation and organ function accurately describing the systemic exposure to aciclovir. Post‐natal age, race and co‐medication use were also tested as potentially influential covariates but were not found to be statistically significant. Inter‐individual variability (IIV) estimates for CL (75.5%) and V (95.3%) were larger than values previously observed in adults and older pediatric patients. Such interindividual variability is likely to reflect the changes associated with renal maturation, and overall organ function, as determined by GFR.^41^, ^42^ During this analysis it also became evident that disease severity (e.g., systemic infection) may alter drug distribution and should be given careful consideration. In addition, our results show that glomerular filtration, as assessed by CL_CR_ corresponds to approximately 25% of the total clearance of aciclovir. This estimate is in line with previously reported data in adults.^43^ In fact, except for a minor contribution of hepatic metabolism (8.5% to 14.1% of total clearance),^44^ studies in which aciclovir was administered together with probenecid and cimetidine have shown that glomerular filtration, tubular secretion by the organic cation transporter and tubular secretion by the organic anion transporter all contribute in equal parts to aciclovir renal elimination.^45^, ^46^ Consequently, in the absence of data relative to tubular secretion, one can assume that the residual clearance (CL_res_) component included in the current model reflects the contribution of both tubular secretion and metabolism.

We acknowledge that in clinical practice, diagnosis of renal dysfunction in pre‐term and term neonates requires more than just monitoring of creatine clearance. Creatinine clearance along with urine output constitute the main domains of the pRIFLE and AKIN criteria, which are used to characterize acute kidney injury (AKI) in critically ill pediatric patients.^47^, ^48^, ^49^ These criteria should be considered when assessing the need for dose adjustment. Regardless of the difficulties in assessing renal function in the period immediately after birth, accurate knowledge of the glomerular filtration rate will be critical for the dose rationale for drugs eliminated by the kidneys. Nephrogenesis in humans is completed by 36 weeks of gestational age, with each kidney comprising 1 000 000 nephrons, with no new nephrons subsequently developing. After this time, the increase in the size of the kidneys is due to the increase in the length and number of cells in existing nephrons. Renal blood flow and glomerular filtration increase during gestation and at 32–35 weeks attain full‐term levels. However, the levels at full‐term are still less than those in adults when corrected for body weight, kidney weight or body surface area.^50^ Likewise, tubular secretion also contributes to the elimination of aciclovir. Tubular function (secretion and absorption) lags glomerular function but still achieves adult levels by 1 year of age.^51^ This tubular immaturity is thought to be due to smaller tubular mass and size as well as less developed active transport processes compared to older children and adults.^52^

An immediate application of this population pharmacokinetic model is the possibility of optimizing and simplifying doses and dosing regimens for the neonatal population, in particular pre‐term newborns.^53^ It enables the simulation of a wider patient population and alternative dosing algorithms. As illustrated here, such an evaluation has shown that aciclovir concentration versus time profiles in preterm neonates receiving the currently recommended dose is lower than in term neonates. Moreover, when comparing these profiles across the different age groups, it appears that the overall exposure in preterm neonates is lower than the levels observed in children, adolescents and adults. Given the well‐known safety profile of aciclovir, and considering the importance of efficacious therapy, further attention should be given to dose recommendations in this vulnerable group of patients.

On the other hand, we recognize that our analysis may have some limitations. First, the limited number of patients contributing to PK data required us to make assumptions about drug disposition, which results in some uncertainty in parameter estimates when extrapolating data. Ideally, physiologically‐based pharmacokinetic (PBPK) modeling may represent a complementary approach to assess the implications of interindividual differences in organ maturation and disease‐related changes in organ function. By relying on a more mechanistic description of the ADME processes, extrapolation using PBPK models may yield more reliable estimates for the pediatric populations. In fact, Jorga et al. showed how population and PBPK modeling has been applied to support the regulatory approval of a dosing algorithm for valganciclovir (VGCV) in children younger than 4 months.^54^ Another issue has been the lack of details on the time course of the viral infection, which would allow a more accurate description of disease severity, and eventually more precise estimates of effect of systemic infection on disposition parameters. In fact, we had to assume that differences in volume of distribution observed between the two studies were determined primarily by varying disease severity, as assessed by positive virological findings. Similarly, the lack of repeated blood sampling for the assessment of pharmacokinetics and renal function during the course of treatment has prevented us from establishing the evaluation of inter‐occasion variability and identification of time‐varying effects, such as inter‐day changes in renal function. Finally, the absence of GFR measurements using gold standard techniques (e.g., inulin, iohexol, 51Cr‐EDTA or Tc99m‐DTPA) required us to use CL_CR_ to describe the relationship between renal function and systemic clearance.

In summary, we have shown that the pharmacokinetics of aciclovir in neonatal patients can be described by a model including parameters that reflect the underlying renal physiology, i.e., considering varying degrees of maturation, and organ function. Albeit not based on a fully mechanistic or physiologically‐based pharmacokinetic modeling approach, our analysis illustrates the impact of empirical dose selection on the systemic exposure to aciclovir in pre‐term and term neonates, as compared to a rationale based on extrapolation principles, which defines a target exposure range associated with efficacy and safety in a reference population.

## AUTHOR CONTRIBUTIONS

SDA performed the analysis and contributed to manuscript writing, DRG contributed to the analysis, ODP contributed to the research objectives, interpretation of results manuscript writing.

## ETHICS STATEMENT

The studies which this analysis was based on were approved by the institutional review boards at each site and were conducted in accordance with the Helsinki Declaration; the legally authorized guardian provided informed consent.

## Supporting information


Data S1.

## Data Availability

The data that support the findings of this study are available from the National Institute of Child Health and Human Development (NICHD). Restrictions apply to the availability of these data, which were used under a data sharing agreement for this study.
