# Supplementary material for: Population pharmacokinetics and dose rationale for aciclovir in term and pre‐term neonates with herpes
Source: Pharmacol Res Perspect. 2024 May 22;12(3):e1193. doi: 10.1002/prp2.1193 (PMC11110484; doi:10.1002/prp2.1193)
Supplement: Supplementary file 1 — Data S1. [file PRP2-12-e1193-s001.pdf]

Supporting Information

Figure S1. Goodness-of-fit plot

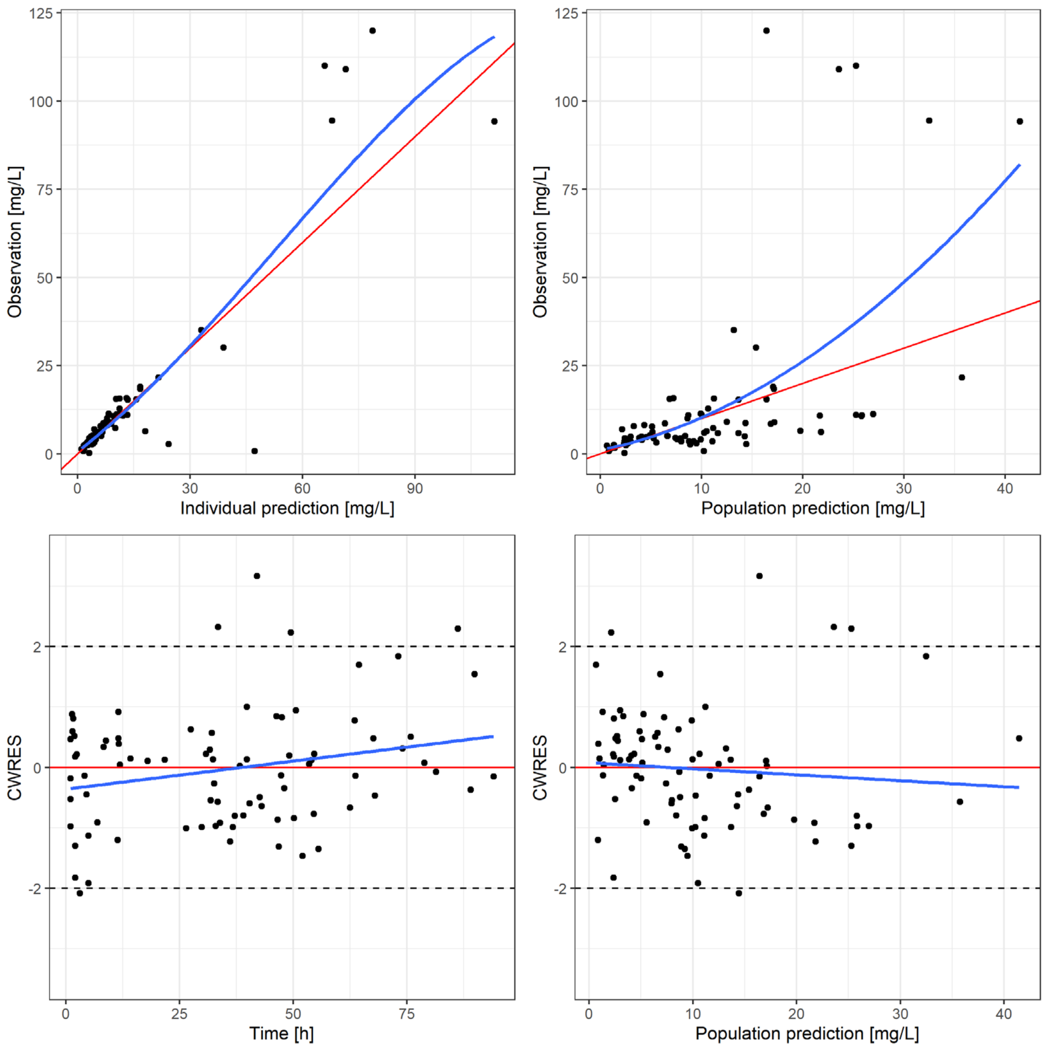

Panels show the goodness-of-fit plots for the final model. Circles represent individual observations/predictions. Blue line is a general linear model smoothing function. Population and Individual prediction refer to population and individually predicted concentrations, respectively. Red lines in the top panels represent the identity line. Red lines in the bottom panels represent the x-axis line.

Figure S2. Mirror plots

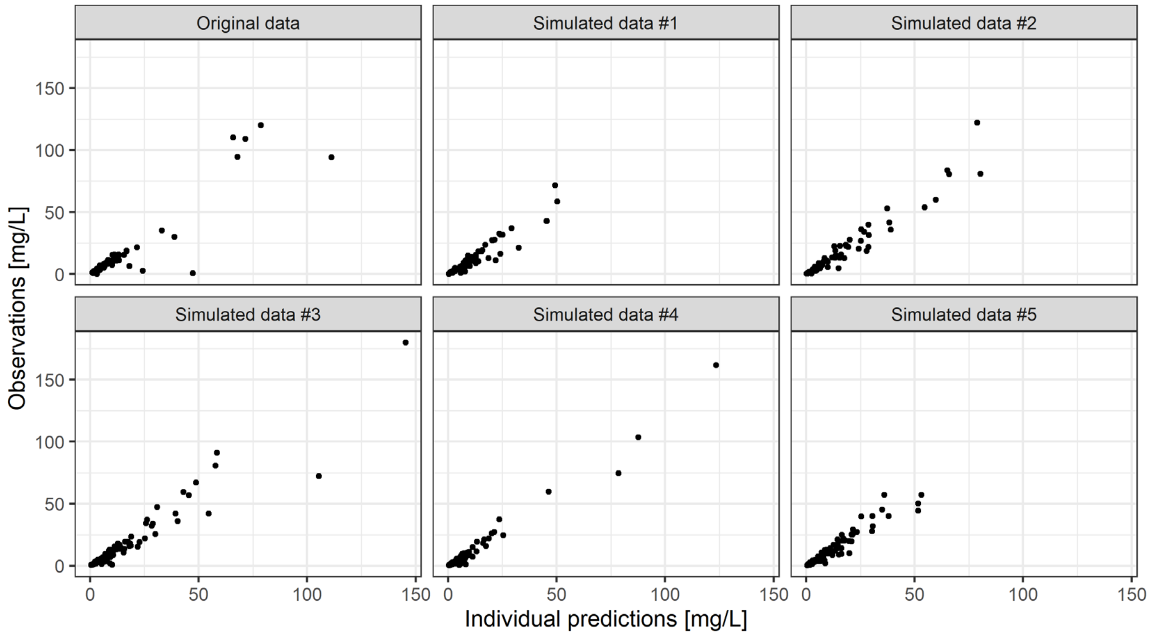

Panels show the final model individual predictions versus observations (left panel) and simulated concentrations vs. predictions (right panels). Circles indicate individually observed/simulated values.

Figure S3. Normalized prediction distribution error (NPDE)

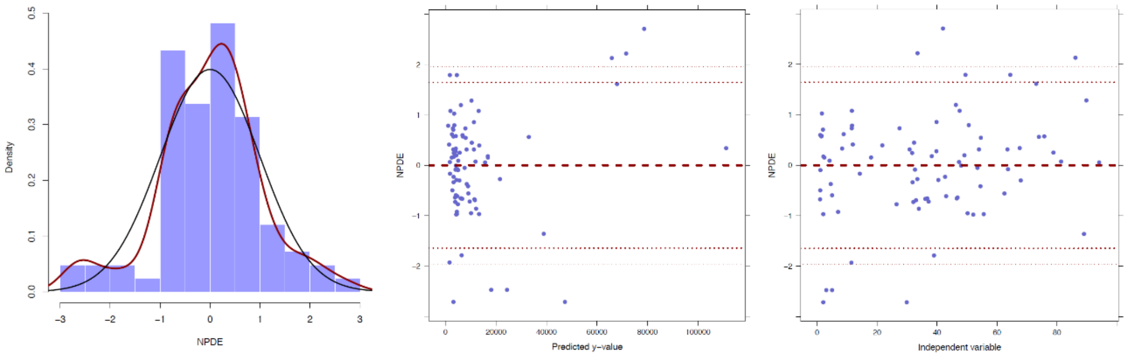

Panels show the NPDE results. Left: Histogram of the distribution of the NPDE, with the density of the standard Gaussian distribution overlaid. Centre: NPDE versus time. Right: NPDE versus population predicted concentrations.

Figure S4. Model performance as assessed by non-standardized visual predictive checks

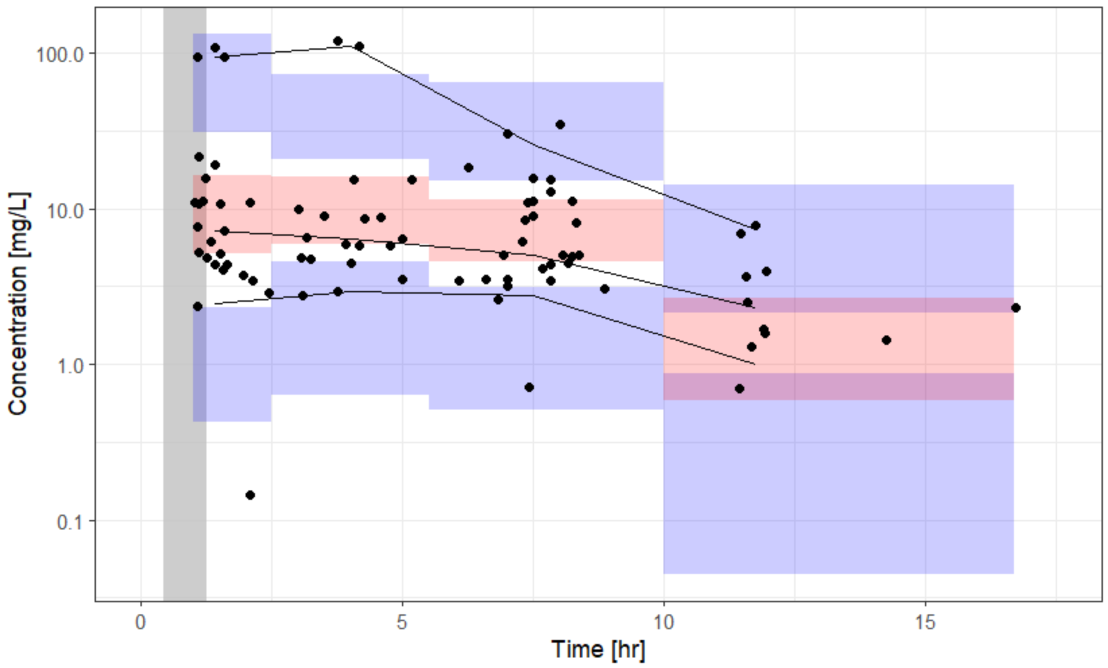

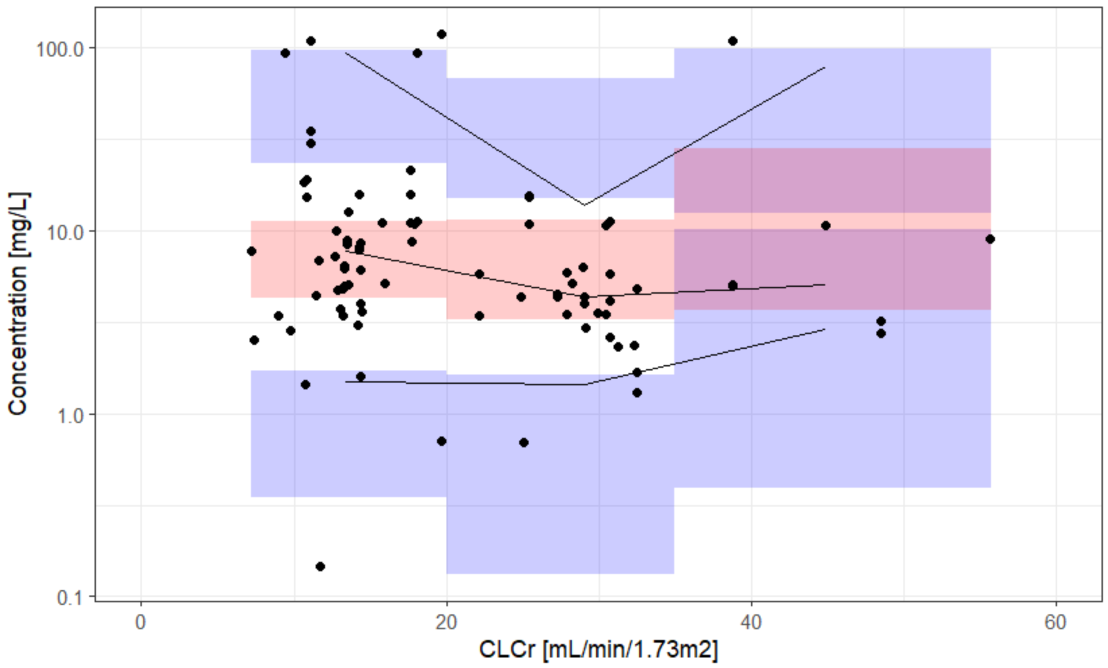

Upper panel: Non-standardized visual predictive check describing model predicted and observed concentration vs time profiles. Grey shaded area depicts the interval between the minimum (0.43 hr) and maximum (1.25 hr) observed infusion durations. Lower panel: visual predictive check showing the lack of correlation between aciclovir concentration and CL_{CR}. In fact, differences in renal function specifically affect only the clearance of aciclovir. Solid line depicts the median, 5th and 95th percentiles of the observed concentrations. Shaded blue and red areas represent the 95%-confidence intervals of the median, 5th and 95th percentiles of the model predicted concentrations. Dots are observed aciclovir concentrations.

Table S1. Summary of Aciclovir Studies Included in the Population Pharmacokinetic Analysis *(ClinicalTrials.gov NCT00942084)

**Study Description**	**Study Population**	**Inclusion criteria**	**Exclusion criteria**	**IV Dose**	**Regimen**	**No. of Evaluable Subjects**	**PK Sampling Scheme**
(Study 1) A single-centre, open-label, PK study of infants 23–42 weeks GA and <61 days PNA	Infants 23–42 weeks GA and <61 days PNA	Infant <4500g and ≤60 days old at the time of initial study drug administration. Suspected to have systemic infection, with appropriate cultures (blood +/- urine/CSF) obtained within 48 hours of study entry. Urine and CSF cultures are based on clinical index of suspicion, but are not required for enrolment; or receiving aciclovir as part of standard of care	• Participants with a history of anaphylaxis attributed to aciclovir<br>• Renal dysfunction indicated by serum creatinine >1.7 mg/dL<br>• Previous participation in the study<br>• Any other concomitant condition, which in the opinion of the investigator would preclude a participant's participation in the study<br>• Infants >4500g and >60days old at time of initial study drug administration	500 mg/m^2	t.i.d.	13	Upon end of first infusion, samples were collected within 5 min, at 2–4 h and at 6–8 h after dose. Upon end of infusion at steady state (doses 5–15), samples were collected within 5 min, at 2–4 h, 6–8 h and immediately before the next dose.
(Study 2) A multi-centre, open-label, PK study of infants 23–34 weeks GA and <45 days PNA	Infants 23–34 weeks GA and <45 days PNA	Infants < 45 days of age at the time of initial study drug administration. Suspected HSV sepsis or two of the following:<br>• Signs of sepsis AND negative blood cultures for >24 hours<br>• Respiratory distress<br>• Lethargy<br>• Fever ≥ 38.0°C<br>• Skin lesions<br>• Seizures (clinical OR EEG confirmed)<br>• Irritability<br>• AST OR ALT >2 X upper limit of normal<br>• >20 WBCs/µL or >500 RBCs/µL	• History of anaphylaxis attributed to aciclovir<br>• Serum creatinine >1.7 mg/dL<br>• Urine output <0.5 mL/kg/hour over the previous 12 hours<br>• Previous participation in the study<br>• Concomitant condition, which in the opinion of the investigator would preclude a participant's participation in the study.	10 mg/kg<br>20 mg/kg<br>20 mg/kg	b.i.d.<br>b.i.d.<br>t.i.d.	19	Upon end of the first infusion, samples were collected within 15 minutes and within 30 min before the second dose. Upon end of infusion at steady state (doses 5–15), samples were collected within 15 min at 2–3 h and within 30 min before the next dose. After the end of the last infusion, samples were collected at 15–18 h.

* Clinical study report available at https://dash.nichd.nih.gov/study/15961

Table S2. Final model and bootstrap results reported by [Sampson et al., 2014]. The final model was a one compartment model including the effect of post-menstrual age on clearance and weight on both clearance and volume of distribution

Parameter	Point Estimate	%RSE	Bootstrap Median (95%CI)
**CL_{pop} (L/h/kg)***	0.305	13.9	0.307 (0.237 – 0.379)
**V_{pop} (L/kg)†**	2.80	14.8	2.80 (1.82 – 3.67)
**θ_{PMA}**	3.02	11.5	3.02 (2.39 – 4.18)
**Interindividual variability (CV%)**			
**CL**	52.8	36.2	53.2 (35.6 – 84.4)
**V**	85.0	51.5	81.3 (4.89 – 140)
**CL-V correlation coefficient**	0.98	45.7	1.00 (0.62 – 1.02)
**Proportional residual variability (CV%)**	34.5	35.0	32.0 (21.1 – 43.3)

*CL (L/h/kg) = CL_{pop} * $(PMA/31.3)^{\theta PMA}$

†V (L/kg) = V_{pop}

Table S3. Final model and bootstrap results reported by [Zeng et al., 2009]

**Parameter (unit)[a]**	**Notation**	**Population Estimate**	**RSE (%)**	**Bootstrap Mean**	**Bootstrap (95% CI)**
Systemic clearance, CL(L/h) = θ1*(WT/19.6)^0.75*(CLCR/3.7)^θ3*EXP(IIV+IOV)	θ1	3.55	5	3.56	3.22–3.94
	θ3	0.51	27	0.49	0.19–0.80
Central volume of distribution, V(L) = θ2*(WT/19.6)*EXP(IIV+IOV)	θ2	7.36	8	7.39	6.25–8.64
k_a = θ4*EXP(IIV)	θ4	0.63	15	0.66	0.46–0.95
F = θ5*EXP(IIV)	θ5	0.6	13	0.6	0.45–0.77
**Inter-individual variability[b]**		**Population Estimate (CV%)**	**RSE (%)**	**Bootstrap Mean**	**Bootstrap (95% CI)**
ηCL variance	Ω1	23.6	30	23	13.9–30.1
ηV variance	Ω2	35.9	31	34.6	21.2–45.8
ηk_a variance	Ω3	58.1	44	57	27.9–84.4
ηF variance	Ω4	41.8	33	39.8	20.8–53.6
**Inter-occasion variability[b]**		**Population Estimate (CV%)**	**RSE (%)**	**Bootstrap Mean**	**Bootstrap (95% CI)**
ηCL variance	Ω5	19.2	32	19.1	12.2–25.0
ηV variance	Ω6	30.4	38	30.8	19.1–42.8
**Residual error**		**Population Estimate (CV%)**	**RSE (%)**	**Bootstrap Mean**	**Bootstrap (95% CI)**
Proportional error	σ1	19.2	32	19.1	12.2–25.0
Additive error	σ2	30.4	38	30.8	19.1–42.8

Abbreviations: CI = confidence interval; CV = coefficient of variation, RSE = relative standard error, WT = body weight, CRCL = creatinine clearance, θ = PK parameter estimation; η = inter-individual variability; Ω = inter-individual or inter-occasion variability in population PK parameter; σ = population variance.

a. Population parameter point-estimates for the full one compartment model and 95% CI and %CV from a non-parametric bootstrap are presented.

b. Value in parentheses represents the inter-individual variability of the PK parameters calculated as the square root of (e^{Ω} – 1) x 100%.

Table S4. Aciclovir exposure in pre-term and term neonates

Reference	Population	n	IV dose[a] (mg/kg) over 1 h	Daily dose[b] (mg/kg)	$C_{min,ss}$ (mg/L)	$C_{max,ss}$ (mg/L)
**Blum 1982**	Adults with normal renal function	24	2.5-15 q8	7.5-45	0.2-2.3	5.1-23.6
**Yeager 1982**	Premature infants PNA<61	4	5-15	15-45	0.34-23.9	5.1-33.7
**Englund 1991**	Neonates with renal dysfunction	16	5-13	5-39	1.2-31.1	5.2-52
**Sampson 2014**	PMA <30 weeks	13	500 mg/m^2 q8 or 10 q12 or 20 q12	1500 mg/m^2 or 20-40	3.92 (2.4-39.3)	10.3 (4.6-110)
	PMA 30-36 weeks	9	500 mg/m^2 q8 or 10 q12 or 20 q12 or 20 q8	1500 mg/m^2 or 20-60	5.1 (2.5-9.6)	8.83 (5.4-29.8)
	PMA 36-41 weeks	6	500 mg/m^2 q8	1500 mg/m^2	2.9 (2.2-7.5)	12.4 (10.8-86.1)
**Current investigation**	PMA <30 weeks	13	500 mg/m^2 q8 or 10 q12 or 20 q12	1500 mg/m^2 or 20-40	4 (0.4-33.6)	7.6 (4.1-119.2)
	PMA 30-36 weeks	9	500 mg/m^2 q8 or 10 q12 or 20 q12 or 20 q8	1500 mg/m^2 or 20-60	6.7 (2.5-46.3)	16.1 (5.5-138.8)
	PMA 36-41 weeks	6	500 mg/m^2 q8	1500 mg/m^2	3.7 (2.6-8)	12.9 (10.4-86.2)

Comparison between previously published data and *post-hoc* estimates from current analysis. Values are range or median (range). a) doses are indicated in mg/kg unless stated otherwise.

Table S5. *Post-hoc* estimates of total aciclovir clearance and calculated CL_{CR} [median (90%CI) mL/min/1.73m^2] for neonatal patients stratified by CL_{CR} and PMA groups (n=28)

	**No. SUBJECTS***	**TOTAL CL (mL/min/1.73m^2)**	**CL_{CR} (mL/min/1.73m^2)**
**CL_{CR} (mL/min/1.73m^2)**			
**0 - ≤10**	4	38.11 (27.71-56.6)	9.02 (7.25-9.72)
**>10 - ≤25**	19	67.02 (24.6-167.28)	13.57 (10.78-20.78)
**>25 - ≤50**	11	151.03 (45.86-290.28)	29.94 (25.39-45.2)
**>50**	1	229.17	55.67
**PMA (weeks)**			
**25 - ≤30**	13	60.95 (26.22-81.44)	13.11 (7.97-32.43)
**>30 - ≤35**	9	141.33 (72.75-234.16)	17.68 (12.78-29.44)
**>35 - ≤41**	7	211.4 (20.88-297.2)	30.43 (18.84-52.05)

**Post-hoc* estimates were calculated taking into account the effect of time-varying covariates during the course of treatment. For this reason, the same subject may be included across different strata.
